# *N*-^11^C-Methyl-Dopamine PET Imaging of Sympathetic Nerve Injury in a Swine Model of Acute Myocardial Ischemia: A Comparison with ^13^N-Ammonia PET

**DOI:** 10.1155/2016/8430637

**Published:** 2016-01-31

**Authors:** Weina Zhou, Xiangcheng Wang, Yulin He, Yongzhen Nie, Guojian Zhang, Cheng Wang, Chunmei Wang, Xuemei Wang

**Affiliations:** ^1^Department of Nuclear Medicine, Affiliated Hospital of Inner Mongolia Medical University, Hohhot, Inner Mongolia 010050, China; ^2^Department of Anesthesiology, Affiliated People's Hospital of Inner Mongolia Medical University, Hohhot, Inner Mongolia 010020, China

## Abstract

*Objective*. Using a swine model of acute myocardial ischemia, we sought to validate* N*-^11^C-methyl-dopamine (^11^C-MDA) as an agent capable of imaging cardiac sympathetic nerve injury.* Methods*. Acute myocardial ischemia was surgically generated in Chinese minipigs. ECG and serum enzyme levels were used to detect the presence of myocardial ischemia. Paired ^11^C-MDA PET and ^13^N-ammonia PET scans were performed at baseline, 1 day, and 1, 3, and 6 months after surgery to relate cardiac sympathetic nerve injury to blood perfusion.* Results*. Seven survived the surgical procedure. The ECG-ST segment was depressed, and levels of the serum enzymes increased. Cardiac uptake of tracer was quantified as the defect volume. Both before and immediately after surgery, the images obtained with ^11^C-MDA and ^13^N-ammonia were similar. At 1 to 6 months after surgery, however, ^11^C-MDA postsurgical left ventricular myocardial defect volume was significantly greater compared to ^13^N-ammonia.* Conclusions*. In the Chinese minipig model of acute myocardial ischemia, the extent of the myocardial defect as visualized by ^11^C-MDA is much greater than would be suggested by blood perfusion images, and the recovery from myocardial sympathetic nerve injury is much slower than the restoration of blood perfusion. ^11^C-MDA PET may provide additional biological information during recovery from ischemic heart disease.

## 1. Introduction

Cardiovascular disease is the leading cause of death worldwide [1], and myocardial ischemia and myocardial infarction are the major causes of fatal heart failure. Early diagnosis of myocardial ischemia and myocardial infarction is required to reduce mortality, particularly through evaluation of electrophysiological changes after cardiac injury. Studies have shown that myocardial ischemia is followed by reduced perfusion and abnormalities of innervation, metabolism, and wall motion, as well as endothelial dysfunction [2–7]. The cardiac sympathetic nerves play a key role in regulating heart function [8]. It has been demonstrated in severe coronary artery stenosis that sympathetic tissues are more susceptible to ischemia than myocardial muscle cells [9, 10]. Accordingly, we hypothesized that cardiac sympathetic nerve injury may be a more sensitive marker of postischemic cardiac damage than changes in blood perfusion during early stage coronary artery events. Many studies have explored myocardial reperfusion and metabolism. However, sympathetic nerve injury and reinnervation, particularly in regard to early diagnosis, have been less examined.

Several compounds have been used for sympathetic nerve receptor imaging in preclinical or clinical studies [11–16]. Both ^123^I metaiodobenzylguanidine (^123^I-MIBG) and ^11^C-hydroxyephidrine (^11^C-HED) are norepinephrine analogs [17, 18], which are accumulated via the norepinephrine transporter (NET) [19]. However, neither tracer has been found to be suitable for the early detection of sympathetic denervation [20]. 6-^18^F-Fluorodopamine (^18^F-DA) is an imaging agent used for the evaluation of primary or secondary lesions in sympathetic tissue [21, 22]. However, the synthesis of ^18^F-DA is expensive and produces low yields, and these problems have limited its use.

We have recently synthesized a novel tracer* N*-^11^C-methyl-dopamine (^11^C-MDA) for cardiac sympathetic nerve imaging. The biological properties of ^11^C-MDA were evaluated in normal mice and healthy Chinese minipigs [23]. ^11^C-MDA had a high myocardium uptake in biodistribution studies. It could be clearly imaged in the heart by PET/CT, and this uptake was blocked by imipramine hydrochloride. Therefore, ^11^C-MDA is a promising candidate radiotracer for imaging the cardiac sympathetic nervous system.

In this study, we established a model of acute myocardial ischemia in Chinese minipigs. Animals were imaged by PET/CT with ^11^C-MDA and ^13^N-ammonia for myocardial blood perfusion. In this way, we were able to observe cardiac sympathetic nerve injury and recovery over a period of 6 months.

## 2. Materials and Methods

### 2.1. Synthesis of ^11^C-MDA and ^13^N-Ammonia

^11^C-MDA and ^13^N-ammonia were synthesized in-house in our PET center cyclotron facility as previously described [23]. Radiochemical purity was greater than 95% for both ^11^C-MDA and ^13^N-ammonia. Radiopharmaceuticals were passed through a 0.2 *μ*m membrane filter for in vivo use.

### 2.2. Animal Model of Cardiac Disease

The study reported here, as well as all aspects of animal maintenance and welfare, was approved by the Inner Mongolian Medical University Animal Care and Use Committee. Ten Chinese minipigs (Shihuang Shiji Mini-Pig Breeding Base, Beijing, China) were maintained in our large animal facility, housed singly, and supplied with the standard diet for swine.

**Figure 1:**
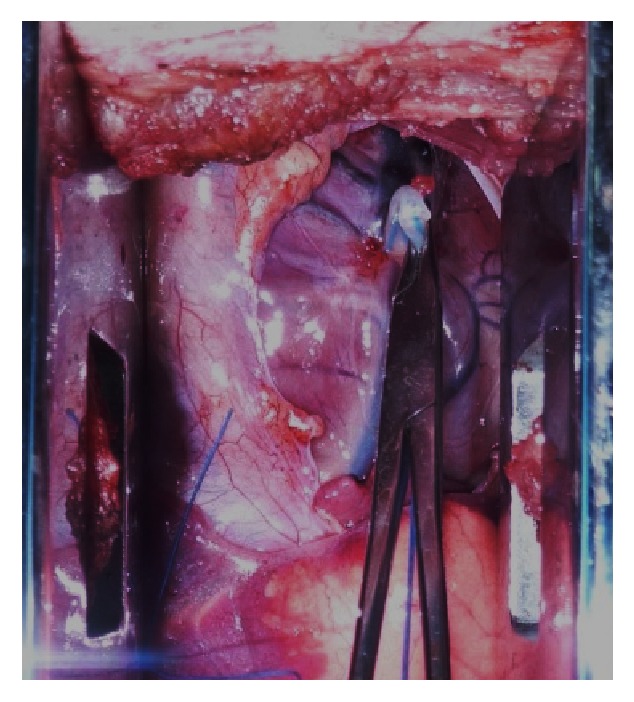
A thoracotomy with blocking of the left anterior descending coronary artery, to generate acute myocardial ischemia in a Chinese minipig.

Surgery was performed as follows: Animals were fasted for 6 hours and intramuscularly injected with ketamine (20 mg/kg) and diazepam (2 mg/kg) for anesthesia; an intravenous route was established at the ear vein and propofol (2 mg/kg/h) was infused to maintain anesthesia. Pigs were placed on the operating table and connected to ECG and oxygen monitor. After successful intubation, the animal underwent ventilator-assisted breathing (tidal volume 8–10 mL/kg, respiratory rate 12–15 beats/min, and respiratory ratio of 1 : 1.5). Hair on the chest was shaved, and the skin was disinfected with 0.1% benzalkonium bromide solution. As shown in Figure 1, a direct thoracotomy was performed at the fifth intercostal space. A 3 mm diameter pneumatic coronary occluder was placed around the left anterior descending coronary artery below the second diagonal branch.

The onset of ischemia was identified by an increase or decrease in the ECG-ST segment of 0.2 mV. Coronary artery occlusion was then released to form acute myocardial ischemia model. Intraoperative electrocardiographic monitoring and venous blood serum enzyme examination (aspartate amino transferase, lactate dehydrogenase, creatine kinase, myocardial muscle creatine kinase, and hydroxybutyrate dehydrogenase) at 9–12 hours after surgery were conducted in all subjects.

### 2.3. PET/CT Image Acquisition

Chinese minipigs were fixed supinely in a special wooden frame and anesthetized as described above. Animals were injected with ^11^C-MDA (74 MBq) and, 10 minutes later, given a 20-minute thoracic PET scan followed by CT. Six hours later, animals received intravenous ^13^N-ammonia (555 MBq) and, after 5 minutes, a 20-minute thoracic PET scan. A clinical PET/CT scanner (DST16-PET/CT, GE Healthcare) was used for 2D nongated PET acquisition, and low dose CT was always performed for attenuation correction (tube voltage 120 kV, tube current 80 mA, rotation time 0.5 s, 1.2 pitch, and thickness 3.75 mm) before each PET scan.

### 2.4. PET/CT Image Reconstruction and Image Analysis

After acquisition, images were transferred to Xeleris (version 4.1, GE Healthcare, USA) and AW (version 4.4, GE Healthcare, USA) image processing workstation for image interpretation and semiquantitative analysis. All image reconstruction was conducted by Butterworth filter function, and the short axis, horizontal long axis, and vertical long axis of the three cross-sectional images were presented.

### 2.5. Statistical Analysis

Data are presented as the mean ± SD. A paired* t*-test was used to compare the effect of treatment on the same imaging agent and an unpaired* t*-test was used to compare the results of two imaging agents. ANOVA was used to analyze the cardiac defect volumes of both imaging agents. SPSS13.0 software (IBM) was used for statistical analysis. A *P* value less than 0.05 was considered statistically significant.

## 3. Results

### 3.1. ECG and Myocardial Enzymes Changes Demonstrate the Induction of Acute Myocardial Ischemia in the Animal Model

**Figure 2:**
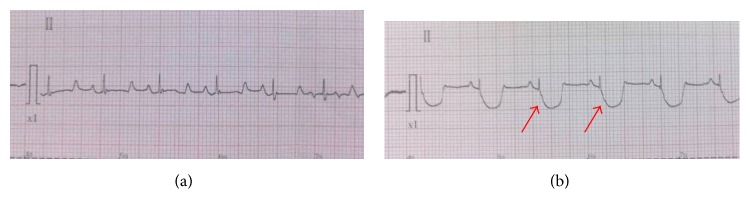
ECG records. (a) Before surgery. (b) After surgery. ST-T segments, showing characteristic depression, are indicated by arrows.

Of the ten pigs that underwent surgery, 7 survived and were available for 6 months of follow-up. Evidence of acute myocardial ischemia was present in all 7 animals. The ECG-ST segment was depressed by over 0.2 mV at lead II (Figure 2). Blood serum enzymes (listed in Table 1) were examined 9–12 hours after surgery, and, for all enzymes examined, all animals showed a marked increase over baseline.

### 3.2. ^11^C-MDA and ^13^N-Ammonia Imaging of Damage to Cardiac Tissue

**Figure 3:**
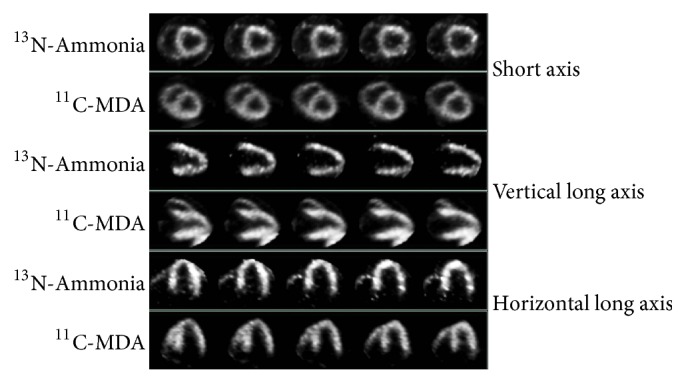
Baseline ^11^C-MDA and ^13^N-ammonia PET in a preoperation pig. ^11^C-MDA and ^13^N-ammonia PET scans were performed over a 6-hour interval. Short axis and vertical and horizontal long axis imaging of the myocardium are presented.

**Figure 4:**
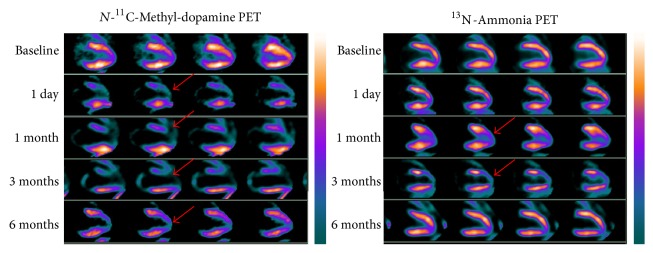
Serial ^11^C-MDA and ^13^N-ammonia PET imaging, shown in the vertical long axis of an injured pig at 1 day, 1 month, 3 months, and 6 months after surgery. The defective region is broader in ^11^C-MDA images compared to ^13^N-ammonia (arrows).

**Figure 5:**
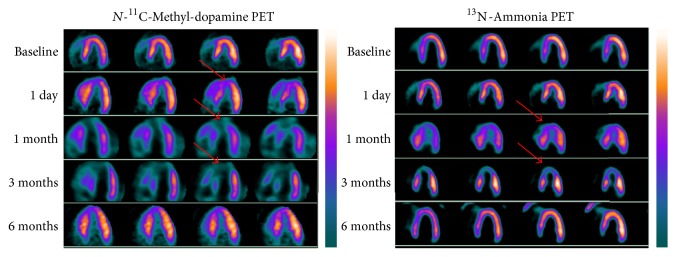
Serial ^11^C-MDA and ^13^N-ammonia PET imaging, shown in the horizontal long axis of an injured pig at 1 day, 1 month, 3 months, and 6 months after surgery. The defective region is broader in ^11^C-MDA images compared to ^13^N-ammonia (arrows).

**Figure 6:**
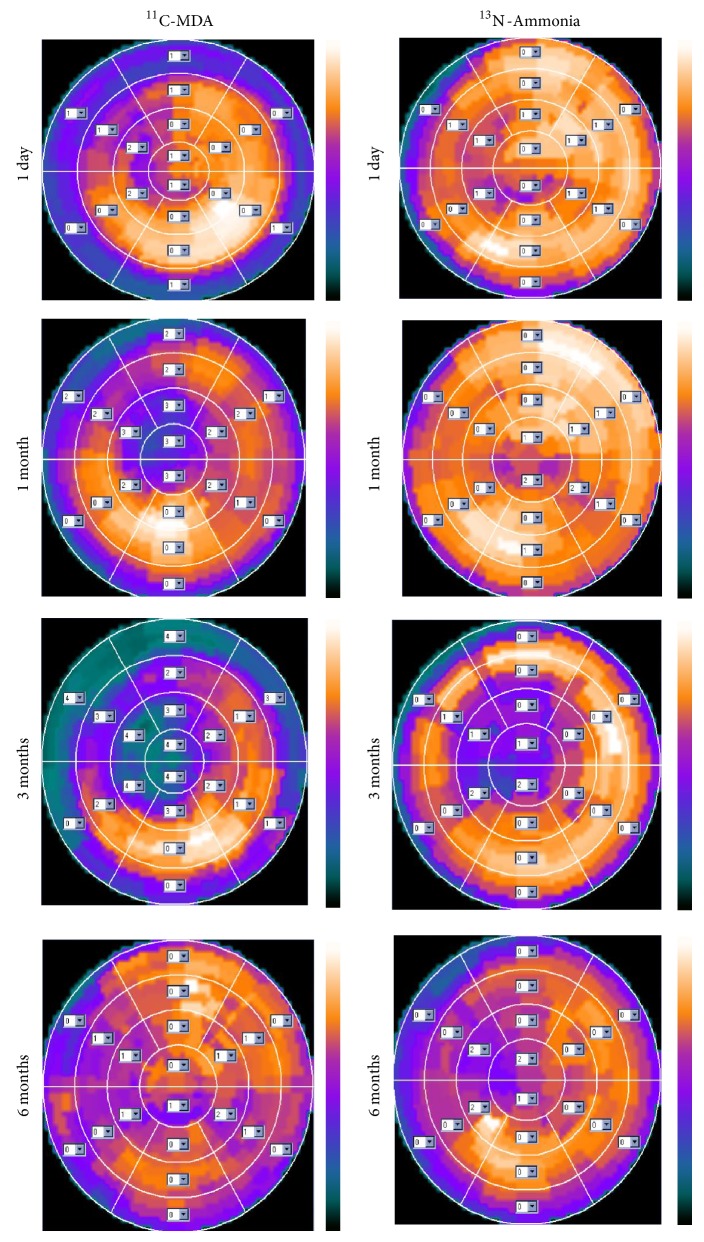
Bull's-eye diagram of ^11^C-MDA and ^13^N-ammonia PET during the 6 months following treatment.

Baseline ^11^C-MDA PET showed uniform distribution of radioactivity in the left ventricular myocardium and mild uptake in the right ventricular wall, which was broadly similar to ^13^N-ammonia myocardium images (Figure 3). Serial ^11^C-MDA and subsequent ^13^N-ammonia scans were performed 1 day and 1, 3, and 6 months after surgery. One day after the surgery, there was obvious uptake defect in ^11^C-MDA imaging but not ^13^N-ammonia; the myocardial uptake defect was the widest in 3 months for both ^11^C-MDA and ^13^N-ammonia. A total restoration of ^13^N-ammonia uptake was observed at 6 months, but recovery for ^11^C-MDA uptake was still on its way. The ^11^C-MDA PET images revealed a defect area that was wider than that suggested by ^13^N-ammonia PET. This was true at all time points examined, as ^11^C-MDA imaging revealed that the defect area recovered slowly (Figures 4 and 5, Table 2), a finding confirmed by the bull's-eye diagram (Figure 6).

## 4. Discussion

In this study, we established acute cardiac ischemia model in Chinese minipig (Figures 1 and 2); we have found that ^11^C-MDA imaging was more sensitive than ^13^N-ammonia for detection myocardium damage following acute ischemic event (Figures 3–6). Unfortunately, we were unable to directly compare ^11^C-MDA and ^123^I-MIBG in this study, owing to the absence of ^123^I in China, and cardiac imaging with ^131^I-MIBG proved ineffective. However, our findings with ^11^C-MDA are consistent with other tracers in clinical use. For example, in a study of 31 patients with coronary heart disease, Hartikainen et al. found that the defect area revealed by ^123^I-MIBG and ^99m^Tc-sestamibi (^99m^Tc-MIBI) imaging was greater than that suggested by myocardial blood perfusion imaging [24]. In 8 patients with multivessel coronary heart disease but without myocardial infarction, Bülow et al. found significantly reduced ^11^C-HED retention, while ^13^N-ammonia was unperturbed [25]. Accordingly, ^11^C-MDA provides more information than blood perfusion imaging for evaluation of post-acute ischemia induced myocardium injury and recovery.

Damage to nervous tissue is an inevitable consequence of myocardial ischemia or infarction, and activation of sympathetic reflex leads to increased vasospasm. As the nutrient supply is restricted and metabolic waste products cannot be efficiently cleared, further nerve damage ensues. Moreover, ATP pools in nerve terminals become depleted, also triggering damage to the nerve endings, and this damage is generally beyond repair. Reperfusion and calcium release from endothelial cells also contribute to damage.

The study also found that myocardial ischemia-reperfusion and cardiac sympathetic restoration occurred over different time scales (Figures 4–6). This finding was consistent with many other studies [26, 27], which showed that animals that had acute myocardial infarction would have varying degrees of cardiac sympathetic nerve regeneration. In 10 patients followed by ^18^F-DA imaging, Fallen et al. [28] found that tracer uptake increased by 14%-15% between 2 weeks and 3 months after myocardial infarction, with no further changes being noted over the next 3 months. Our results are thus in line with previously published data, bearing in mind the different experimental model and the difference between myocardial ischemia and myocardial infarction. The mismatch between the restoration of myocardial perfusion and normal myocardial sympathetic response could cause life-threatening ventricular arrhythmias [29], and at the same time immune reactions were more likely to occur to harmful ventricular arrhythmias [30, 31]. Sympathetic nerve regeneration plays a critical role in ventricular arrhythmias [32], and in general cardiac pathology [33], including sudden cardiac death, congestive heart failure, and diabetic autonomic neuropathy [34, 35]. Our study found that the use of ^11^C-MDA PET/CT imaging could successfully detect abnormal sympathetic tissue in the damaged heart (Figures 4 and 5), and this agent may have an important role in cardiac imaging.

The objective of this study was to compare ^11^C-MDA PET imaging sympathetic nerve recovery with ^13^N-ammonia for blood perfusion; dynamic PET acquisition immediately after tracer inoculation was not conducted in this study. Dynamic PET data analysis for compartment modeling was not performed in this study, and a future study may need to focus on this issue.

## 5. Conclusion

Using a Chinese minipig model of acute myocardial ischemia, we have demonstrated that ^11^C-MDA is capable of imaging cardiac damage and provides information that is distinct from blood perfusion imaging. ^11^C-MDA PET may provide additional biological information during recovery from ischemic heart disease.

## Figures and Tables

**Table 1:** Serum levels of myocardial enzymes before and after surgery.

	AST (U/L)		LDH (U/L)		CK (U/L)		CK-MB (U/L)		HBDH (U/L)	
	Before	After	Before	After	Before	After	Before	After	Before	After
1	20.0	410.9	120.0	1992.0	180.0	23720.0	15.0	664.2	84.5	1617.0
2	18.0	123.1	135.0	978.0	154.8	18083.0	13.4	481.6	79.4	874.0
3	8.0	331.2	158.0	3227.0	198.0	4014.0	7.0	372.7	80.0	1548.0
4	21.0	195.6	181.0	2559.0	148.2	7418.0	14.0	164.7	154.0	822.0
5	30.0	164.6	153.0	1579.0	75.0	8185.0	6.9	265.2	135.4	1657.0
6	25.3	215.7	127.5	1375.0	176.0	5224.0	10.7	273.5	92.7	1385.0
7	27.8	305.5	159.3	1498.0	210.0	6718.0	13.2	318.7	127.4	1439.0

Mean	21.4	249.5	147.7	1886.9	163.1	10480.3	11.5	362.9	107.6	1334.6
SD	7.3	102.5	21.3	773.8	44.6	7431.2	3.3	165.2	30.6	345.8

AST: normal reference value (8–37 U/L); LDH: normal reference value (110–240 U/L); CK: normal reference value (0–250 U/L); CK-MB: normal reference value (0–25 U/L); HBDH: normal reference value (72–182 U/L).

**Table 2:** Comparison of defect scores, ischemic-to-normal ratios, and volume of ischemic myocardium for ^11^C-N-CH_3_-dopamine and ^13^N-ammonia PET imaging at each time point after myocardial ischemia-reperfusion.

Time	Defect score			Ischemic-to-normal ratio			Volume of ischemic myocardium (cm^3^)		
	^11^C-N-CH_3_-dopamine	^13^N-Ammonia	*P*	^11^C-N-CH_3_-dopamine	^13^N-Ammonia	*P*	^11^C-N-CH_3_-dopamine	^13^N-Ammonia	*P*
1 day	10.2 ± 2.5	9.9 ± 0.9	NS	0.61 ± 0.12	0.61 ± 0.08	NS	3.79 ± 0.06	3.66 ± 0.08	NS
1 month	13.3 ± 3.3	7.7 ± 1.0	<0.001	0.51 ± 0.22	0.60 ± 0.10	<0.05	4.21 ± 0.34	2.54 ± 0.11	<0.001
3 months	18.6 ± 4.4	4.9 ± 1.0	<0.001	0.42 ± 0.13	0.60 ± 0.09	<0.05	10.67 ± 0.71	2.47 ± 0.12	<0.001
6 months	10.7 ± 4.2	4.1 ± 0.9	<0.001	0.60 ± 0.17	0.60 ± 0.09	NS	5.96 ± 0.50	2.43 ± 0.11	<0.001
